# Electromagnetic compatibility of implantable neurostimulators to RFID emitters

**DOI:** 10.1186/1475-925X-10-50

**Published:** 2011-06-09

**Authors:** Oxana S Pantchenko, Seth J Seidman, Joshua W Guag, Donald M Witters, Curt L Sponberg

**Affiliations:** 1 U.S. Food and Drug Administration, 10903 New Hampshire Avenue, Silver Spring, MD 20993, USA; 2 University of California at Santa Cruz, Baskin School of Engineering, 1156 High Street, Santa Cruz, CA 95064, USA; 3 Medtronic Neuromodulation, 7000 Central Ave NE, Minneapolis, MN 55432, USA

## Abstract

**Background:**

The objective of this study is to investigate electromagnetic compatibility (EMC) of implantable neurostimulators with the emissions from radio frequency identification (RFID) emitters.

**Methods:**

Six active implantable neurostimulators with lead systems were tested for susceptibility to electromagnetic fields generated by 22 RFID emitters. These medical devices have been approved for marketing in the U.S. for a number of intended uses that include: epilepsy, depression, incontinence, Parkinsonian tremor and pain relief. Each RFID emitter had one of the following carrier frequencies: 125 kHz, 134 kHz, 13.56 MHz, 433 MHz, 915 MHz and 2.45 GHz

**Results:**

The test results showed the output of one of the implantable neurostimulators was inhibited by 134 kHz RFID emitter at separation distances of 10 cm or less. The output of the same implantable neurostimulator was also inhibited by another 134 kHz RFID emitter at separation distances of 10 cm or less and also showed inconsistent pulsing rate at a separation distance of 15 cm. Both effects occurred during and lasted through out the duration of the exposure.

**Conclusions:**

The clinical significance of the effects was assessed by a clinician at the U.S. Food and Drug Administration. The effects were determined to be clinically significant only if they occurred for extended period of time. There were no observed effects from the other 5 implantable neurostimulators or during exposures from other RFID emitters.

## Background

In the last several years, radio frequency identification (RFID) technology has become a popular choice for tracking people, animals, products and goods. This type of technology serves the same purpose as bar coding systems and magnetic strip systems, which is to provide identification. One advantage of RFID technology versus other types of technologies is the proximity for identification. Another advantage is the information stored in such systems can be programmed and reprogrammed, providing a robust way to store information. For example, bar code scanners (readers) need direct line of sight to identify barcodes. In magnetic strip technology, the magnetic strip cards have to be swiped through or very close to the reader to be identified. In RFID technology, RFID readers can be feet away from identification tags and still be able to identify them. This technology works by emitting and receiving radio and sub-radio frequency electromagnetic energy. Since RFID technology has gained popularity in many industries, an average person could get exposed to the emitted fields from RFID readers when using public transportation, shopping at a grocery store, picking up a package at a postal service and driving through a toll booth [1,2].

RFID technology is favored for certain uses due to its contactless transfer of data and storage capacity [1] and is quickly merging into health care and pharmaceutical industries. The United States Food and Drug Administration (FDA) is encouraging use of a state-of-the-art technology, such as RFID, that tags product packaging electronically because it allows manufacturers and distributors to precisely track drug products through the supply chain [3]. A number of hospitals are adopting RFID technology to help locate doctors, nurses, patients and expensive medical equipment [4] and RFID systems have been deployed for tracking items used in the surgical suites, such as, sponges, needles and surgical instruments [5]. Other RFID systems have capability to monitor temperature and are being utilized in pharmaceutical industry for temperature compliance purposes [4]. Overall, RFID systems offer a variety of benefits, including fast transactions, real time tracking, contactless data transfer, large storage capacity and continuous temperature monitoring. Some claim that RFID technology can change the delivery of patient care [6].

Similarly to other sources of electromagnetic energy, the emissions from RFID systems can be a source for electromagnetic interference (EMI) with medical devices. The potential risks of EMI with RFID emissions can be illustrated by the study conducted by van der Togt *et. al*. that reported potentially hazardous incidents in critical care medical equipment caused by RFID system emissions. The hazardous incidents were events that could have a direct physical influence on a patient by unintended change in equipment function [7]. In a more recent study, Seidman *et. al*. investigated electromagnetic compatibility (EMC) between RFID and implantable pacemakers and implantable cardioverter defibrillators (ICDs). The pacemakers and ICDs were exposed to RFID readers of 134 kHz, 13.56 MHz, and 915 MHz carrier frequencies. The results showed that during 134 kHz RFID reader emissions, a reaction was observed for 67% of all pacemaker tests and 47% of all ICD tests. Observed reactions by implantable pacemakers and ICDs included pacing inhibition, inappropriate pacing, noise reversion mode, changed pacing rates, inappropriate delivery of antitachycardia pacing, inappropriate delivery of high voltage shocks and a change in device programming [8].

Active implantable neurostimulator devices are similar to pacemakers and ICDs that potentially could be susceptible to electromagnetic interference from RFID emissions. Unlike implantable pacemakers and ICDs, FDA approved implantable neurostimulators do not have sensing capability and operate as open loop systems with the patient to close the loop. Implantable pacemakers and ICDs include cardiac sensing capabilities in order to sense electrophysiological signals that might make these devices more sensitive to external low frequency RF signals. Due to their sensing capabilities, it is hypothesized that pacemakers and ICDs might be more likely to misinterpret certain external RF emissions as an electrophysiological signal.

In recent years, the FDA has received a number of reports suggesting EMI with deep brain stimulators from various electromagnetic sources [9]. Kainz *et. al*. described various sources of EMI, which included a report from a patient with an implantable spinal cord stimulator who received an electric shock while walking near an article surveillance device [10]. Several incident reports and published literature culminated in the 1998, the FDA advisory issued a letter to cardiologists, cardiovascular surgeons, emergency physicians, neurologists and neuro surgeons warning about the operation of certain medical devices, including spinal cord stimulators, may be affected by the electromagnetic fields produced by anti-theft systems and metal detectors [11]. The present study extends the investigation of potential EMI effects on implantable neurostimulators with emissions from RFID emitters.

## Methods

### Materials

Six active implantable neurostimulators were analyzed for EMC with 22 RFID emitters. Each implantable neurostimulator was approved by the FDA for intended uses that include: epilepsy, depression, incontinence, Parkinsonian tremor and pain relief. All six implantable neurostimulators consisted of implantable pulse generators (IPG) and implantable leads with platinum/iridium electrodes. All implantable neurostimulators were open loop systems where the physician and patient program and control the stimulation. During testing exposures to the emissions from the RFID, all neurostimulators were carefully monitored for effects of EMI, such as change in stimulating parameters, changes in programmable settings, change in operating mode, false alarms, initiation of any unintended operation and changes in programmable parameter settings. Due to the nature of this study and the cooperative agreement set up between multiple neurostimulator manufacturers and the FDA, the name and the model of each implantable neurostimulator will be withheld and device under test (DUT) number assigned. Table 1 lists additional characteristics of the tested implantable neurostimulators.

**Table 1:** Characteristics of Implantable Neurostimulators

DUT	Indication	Stimulation Modality	Longest Lead Length Tested (cm) ^a^	Number of leads connected to the neurostimulator
1	Epilepsy and depression	Bi-polar	43	1
2	Epilepsy and depression	Bi-polar	43	1
3	Incontinence	Bi-polar	41	1
4	Parkinsonian tremor	Bi-polar	135	2
5	Pain relief	Bi-polar	75	2
6	Pain relief	Bi-polar	135	2

^a ^In the case of the neurostimulator system tested with lead extensions, the longest length reported includes the length of the extension lead.

Twenty two RFID emitters were used in EMC testing with implantable neurostimulators. LF and HF systems primarily emit magnetic fields while UHF systems primarily emit electric fields. Table 2 shows the appropriate RF and physical characteristics of all 22 RFID emitters. For Emitters 1-5, the measurements were made with magnetic field probe Model 1709.001 (Electric Research and Management) and for Emitters 6-13 the measurement were made with magnetic field probe, Model H3DV7 (SPEAG). For Emitters 14-16 and 21, 22, the measurements were made with electric field probe, Model SRM-3000 (NARDA) and for Emitters 17-20, the measurement was made with electric field probe, Model HI-6105 (ETS-Lindgren). In all measurements, probes were connected to a robotic arm to maneuver along RFID antennas. The probes were aligned perpendicularly to RFID antennas and measured field strength along the flat surface of each antenna.

**Table 2:** Characteristics of RFID emitters

Emitter	Emitter/ Reader Antenna Dimensions (cm)	Standard Used	Emitter Carrier Frequency (MHz)	Maximum H-field (A m^-1^) at 2.5 cm ^a^	Maximum E-field (RMS) (V m^-1^) at 10 cm
1	Rectangular Loop 114 × 66 × 6.3	__	0.125	13.3	__
2	Rectangular Loop 3.5 × 4.5 × 0.5	__	0.125	1.2	__
3	Rectangular Loop 20 × 20 × 2.5	ISO 11785	0.134	269.0	__
4	Rectangular Loop 85 × 50 × 5	__	0.134	68.0	__
5	Rectangular Loop 85 × 50 × 5	ISO 11785	0.134	162.0	__
6	Rectangular Loop 31 × 31 × 2.8	ISO 18000-3 mode 1	13.56	4.6	__
7	Rectangular Loop 20 × 20 × 0.8	ISO 18000-3 mode 1	13.56	4.9	__
8	Rectangular Loop 31 × 31 × 2.8	__	13.56	8.6	__
9	Rectangular Loop 31 × 31 × 2.8	ISO 18000-3 mode 1	13.56	8.7	__
10	Rectangular Loop 31 × 31 × 2.8	ISO 18000-3 mode 1	13.56	8.8	__
11	Handheld 19 × 11 ×7.8	ISO 18000-3 mode 1	13.56	7.8	__
12	Patch 2.3 × 2.5 × 0.1	ISO 1443A, 1443B, 15693, 18000-3	13.56	2.4	__
13	Patch 21 × 32 × 1.2	ISO 18000-3 Mode 2	13.56	18.6	__
14	Patch 15.7 × 5.5 × 3	ISO 18000-7	433	__	0.4
15	Stick length 19.8 × diameter 1.4	__	433	__	___
16	Patch 38 × 36.5 × 1.5	__	433	__	___
17	Patch 31 × 31 × 4.8	ISO 18000-6B	915	__	79.3
18	Patch 48.5 × 31 × 5	ISO 18000-6B	915	__	36.2
19	Patch 21 × 21 × 3.5	ISO 18000-6	915	__	97.3
20	Patch 22.5 × 21 × 5	ISO 18000-6C	915	__	69.8
21	Stick length 10.5 diameter 0.9	__	2450	__	0.02
22	Stick length 11 diameter 0.8	__	2450	__	1.0

^a ^Maximum H-field strength at 2.5 cm for Emitters 1-5 was measured using magnetic probe Model 1709.001, Electric Research and Management. For Emitters 6 - 13, the maximum H-field strength was measured using magnetic probe, Model H3DV7, Speag. For Emitters 14-16 and 21, 22, the maximum E-field was measured using E-field probe, Model SRM-3000, NARDA. Measured electric field of Emitters 15 and 16 was below the sensitivity level of the probe. For Emitters 17-20, the maximum E-field was measured using E-field probe, Model HI-6105, ETS-Lindgren.

In general, RFID systems consisted of interrogators (active emitters) and transponders (active and passive emitters). The interrogators, sometimes called readers, were designed to read information from transponders. RFID transponders are referred to as tags are either active or passive devices that transmit their information to reader. For this study, the term RFID emitter describes the 21 RFID readers and one active RFID tag. Five of the RFID emitters operated using a carrier frequency between 125 and 135 kHz and are typically used for access control for animals and people. Twelve RFID emitters operate using a carrier frequency of 13.56 MHz that is typically used in libraries, passports, payment emitters and smart cards. Three of the tested RFID emitters operated using a carrier frequency of 433MHz that are typically used for asset tracking. One of the tested RFID emitters operated at a carrier frequency of 915 MHz that are typically used in retail and military supply chain tracking. Lastly, one RFID emitter operated at a carrier frequency of 2.45GHz typically limited to niche uses [8]. Due to the nature of this study and the cooperative agreement set up between multiple RFID manufacturers and the FDA, the name and the model of each RFID emitter will be withheld and RFID number assigned.

### Test Method

**Figure 1:**
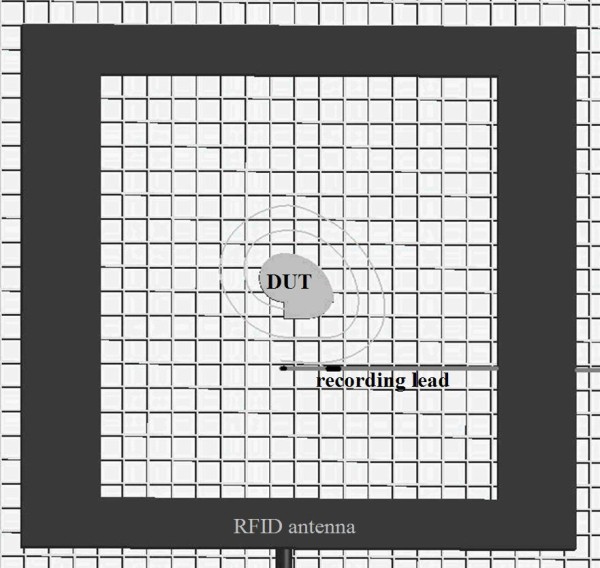
**Overall Configuration**. Configuration of RFID antenna, implantable neurostimulator system and pick-up lead. The implantable neurostimulator system and the pick-up lead were positioned in the same z- plane. The RFID antenna was parallel to the neurostimulator system.

The test method for the present study originated from ANSI/AAMI/ISO 14708-3:2008 American National Standard for Implants for Surgery - Active implantable Medical Devices - Part 3: Implantable Neurostimulators [12]. The standard was recognized for developing *in vitro *EMC test protocols for implantable neurostimulators using a torso simulator tank made of a polyethylene plastic box (58.5 cm × 42.5 cm × 15.2 cm) filled with 0.27 S/m saline solution to represent static electrical properties of the body. A non-conductive, non-metallic plastic grid cut from a fluorescent light fixture cover was used as a support grid for the neurostimulator device and the lead system. The grid was suspended with plastic legs inside the saline solution that allows adjustable elevation within the saline. The DUT was submerged 5 mm deep into the saline bath parallel to the surface. Figure 1 demonstrates top view of the test set-up.

Four out of the six implantable neurostimulators use voltage based stimulation and were programmed to generate pulses of electrical potential amplitude (4.2-5.2 V), frequency (2-3 Hz), pulse width (210-500 usec). The other two implantable neurostimulators use a current based stimulation and were programmed to output pulses of electrical current amplitude (1.75 mA), frequency (2 Hz) and pulse width (500 usec). Overall, the output amplitude of the devices was set to the half of their peak maximum amplitude, the maximum pulse width and the smallest programmable frequency. These parameters were chosen to allow the greatest range of observation for detecting disruptions or changes to the DUT output that could be attributed to RFID emissions exposure.

In the cases where a neurostimulator system had more than two stimulating electrodes per lead system, the electrodes that were furthest apart were activated for consistency among all tested systems. If a neurostimulator system had an option of using bi-polar or unipolar setting, the bi-polar setting was chosen for consistency among all tested neurostimulator systems. If a neurostimulator system had an option of using different lead lengths, the leads of maximum lengths were chosen due to the expectation that this would capture a larger portion of the RFID emissions and allow for greater coupling to the electromagnetic fields into the DUT. A bi-polar pacemaker lead connected to a digital oscilloscope was placed in the saline bath within a few millimeters from the neurostimulator electrodes to record the output pulses from the DUT.

**Figure 2:**
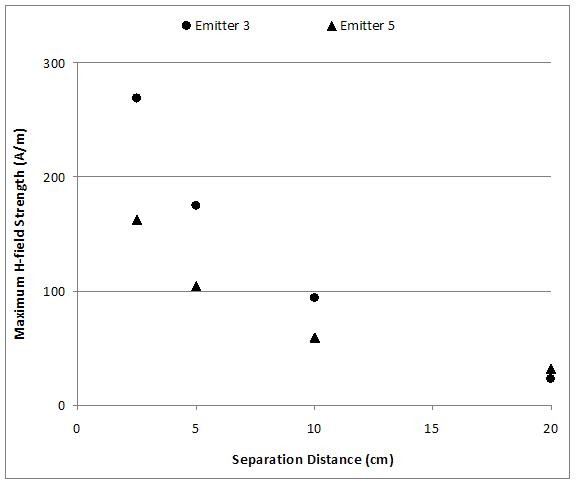
**H-field Measurements**. Measured maximum H-field strength at 2.5, 5, 10 and 20 cm away from the antenna for Emitters 3 and 5.

A non-conductive, non-metallic fiberglass robotic arm was used to maneuver each RFID emitter parallel to the open surface of the stimulator system. Starting at 60 cm vertical separation distance from the DUT, the robotic arm moved the RFID emitter closer to the submerged DUT in increments of 5 cm. The closest separation distance between the DUT and the RFID emitter was 2.5 cm. Figure 2 shows the H-field strengths at perpendicular distances apart from the RFID emitter antennas for Emitters 3 and 5.

### Test Procedure

Each DUT was configured with IPG placed in the middle of the support grid and the leads and electrodes wrapped in a spiral around the IPG as specified in the ANSI/AAMI/ISO 14708-3:2008 Standard [12]. For consistency, each lead was wrapped twice around the IPG with the distance between the center of IPG to the furthest electrode held equal to the following,(1)

The lead system was secured in place with non conductive cotton thread and the conductivity of the saline bath measured and if needed corrected to 0.27 S/m. The DUT with the support grid was submerged into the saline tank to a depth of 5 mm. The bi-polar recording lead was then placed into the saline bath within millimeters away from DUT. The DUT was programmed and activated and output of the IPG was verified on the oscilloscope. For each measurement, the robotic arm was raised 60 cm away from DUT then RFID emitter antenna was placed on top of the robotic arm and centered directly over the DUT. The RFID emitter was turned on and verified for proper operation. The robotic arm with RFID antenna were lowered in 5 cm increments until the separation distance of 5 cm was reached. The robotic arm was then additionally lowered to 2.5 cm above the DUT for worst case test. Thirteen distances of separation were verified for exposures. The behavior of the neurostimulator under each test condition was observed for 15 seconds and recorded. Any change in output signal was noted as an effect. The same test procedure was repeated for each of the 22 RFID emitters resulting in the total of 1716 observed tests (6 neurostimulator systems × 22 RFID emitters × 13 tested distances).

## Results

EMC was investigated between six implantable neurostimulators and 22 RFID emitters at 13 distances of separation. A total of 1716 tests were administered. Six tested implantable neurostimulators did not show any effects when exposed to RFID emitters with 125 kHz, 13.56 MHz, 433 MHz, 915 MHz and 2.45 GHz carrier frequencies and continued their normal mode of operation before, during and after exposures.

Seven effects were observed from exposures between RFID carrier frequency of 134 kHz and DUT 3. The output of DUT 3 with lead length of 41 cm was inhibited by Emitter 3, with a 134 kHz carrier frequency, at separation distances of 2.5, 5 and 10 cm. The output of the same implantable neurostimulator with the same lead length was also inhibited by Emitter 5, with a carrier frequency of 134 kHz, at separation distances of 2.5, 5, 10 cm and also showed inconsistent pulsing rate at a separation distance of 15 cm. All effects were transient occurring only during exposure.

In order to investigate EMC of DUT 3 further, additional tests were performed. The neurostimulator was tested with shorter lead lengths of 28 cm and 33 cm. The DUT 3 with each lead length was exposed to Emitters 3 and 5 in the same way as described in the Test Method and Test Procedure sections of this paper. The following effects were observed,

• DUT 3, lead length of 33 cm exposed to Emitter 3

○ the output was inhibited at 2.5, 5, and 10 cm of separation distance

• DUT 3, lead length of 33 cm exposed to Emitter 5

○ the output was inhibited at 2.5 and 5 cm of separation distance

• DUT 3, lead length of 28 cm exposed to Emitter 3

○ inconsistent pulsing rate at a separation distance of 10 cm

○ the output was inhibited at 2.5 and 5 cm of separation distance

• DUT 3, lead length of 28 cm exposed to Emitter 5

○ the output was inhibited at 2.5 and 5 cm of separation distance

All DUT effects occurred during and lasted throughout the duration of the particular separation distance and exposure from RFID emitters. Additionally, Figure 2 shows measured maximum H-field strength at distances of interest.

## Discussion

Effects from EMI were observed when DUT 3 was exposed to the electromagnetic fields from RFID emitters operating at the 134 kHz carrier frequency. Two major distinctions between 134 kHz RFID emitters and emitters of higher frequencies are their carrier frequency and the antenna type. Close to the emitter antennas, also known as the "near field" region, low frequency antennas emit primarily magnetic fields. This is the case for tested 134 kHz RFID tested emitters. Emitters 3 and 5 that caused EMI have magnetic field intensities that are at or above 162 A/m at 2.5 cm away from antenna. From theory, in the near field region, the strength of the magnetic field decreases with the cube of a distance [1]. This explains why the same types of effects did not occur at greater distances of separation.

More effects on the DUT3 were observed at larger distances of separation with Emitter 5 that has H-field strength less than the one of Emitter 3 at 2.5 cm. This could be due to the effects that larger antennas have. It is true that for RFID antennas with larger dimensions, the field strength decreases more slowly than for antennas of smaller dimensions. Figure 2 demonstrates the principal of field strength curve vs. distance of separation. For example, Emitter 3 has higher field strength at the separation distance of 10 cm; however, Emitter 5 has higher field strength at 20 cm of separation distance. This could explain observed effects seen at 15 cm with Emitter 5 and not with Emitter 3.

Additional testing of DUT 3 with lead lengths of 33 cm and 28 cm and Emitters 3 and 5 indicate that the lead length is related to a number of effects. DUT 3 with lead length of 41 cm, seven effects were observed with the maximum distance of separation of 15 cm. DUT 3 with lead length of 28 cm, five effects were observed with maximum distance of separation of 10 cm, therefore the number of observed effects increases with increased lead length. This effect could be due to the induced current coupling into the leads and the neurostimulator system. Induced current is proportional to the area formed by the lead; the larger the area, the greater the electric current in the system.

One possible explanation for the effects seen with DUT 3 and Emitters 3 and 5 is related to the telemetry frequency (frequency used to communicate between the neurostimulator and the programmer) and the neurostimulator control design. The telemetry frequency of DUT 3 is very near carrier frequency of Emitters 3 and 5. Additionally, DUT 3 is designed to inhibit stimulation during telemetry process. This period lasts between 42 - 84 ms depending on the number of ones and zeros in the data. If the RFID reader emits a pulse every 42 ms (or quicker) than we would expect complete inhibition. Depending on timing RFID readers that pulse less frequently could partially inhibit the neurostimulator.

The clinical significance of the effects was analyzed by a clinician at the FDA. Two scenarios were considered, short term exposure and extended period of time exposure from RFID emitters. Short term exposure is equivalent to a patient walking through or by RFID emitter. Short term exposure could cause temporary effects, in this case only minor changes or no changes at all would likely be noted by the patient. Extended period of time exposures are equivalent to a patient spending long periods of time near an RFID emitter. In this case, long term exposure could cause long term effects that are clinically significant and therefore making it likely that the patient's original symptoms of incontinence to return.

## Limitations

There are several limitations of this study that should be discussed. The study was conducted on a phantom filled with saline solution representing average conductivity of a human body at all frequencies of interest. During the study, the RFID antennas were centered over implantable neurostimulator systems, which might not represent the worst case scenario for square or rectangular antennas since the maximum field strength is at the corners. Also, the configuration of implantable pulse generator and the lead system was a generalization across these devices taken from referenced standard [12] rather than a strict adherence to the way each device would likely be implanted. This configuration was chosen because it seems close to the worse case scenario for potentially inducing electromagnetic interference and maximizes the area and number of loops made by the lead system. Another important limitation of this study is the operation of RFID reader without RFID tag. In our study, we used RFID tags to verify proper operation of RFID emitters prior to testing and we did not use RFID tags during dwell time of each test. The presence of an RFID tag could effect the reader's modulation. It is also important to mention that few of our tested implantable neurostimulator systems had an option of unipolar stimulation. As we mentioned in Test Method section, we chose a bi-polar stimulation option for consistency purposes, since all of our neurostimulators offered such option. The unipolar stimulation option was not tested which defines anther limitation of this study.

## Conclusions

In this study, EMC was investigated among six implantable neurostimulators and 22 RFID emitters. Most of the DUT implantable neurostimulators were unaffected by the RFID emissions. However, DUT 3 showed repeatable effects that included output inhibition and inconsistent pulsing rate from exposure to 2 different 134 kHz RFID emitters at separation distances from 2.5 cm up to 15 cm. The present study seems generally consistent with a previously published study of active implantable devices that showed that EMI is most common for certain medical devices during exposure to 134 kHz frequency RFID emitters [8]. Manufacturers of active implantable devices need to be aware of the potential and risk of EMI from RFID emitters and design and their medical devices appropriately. Additionally, RFID industry should take into account the potential effects on active implantable medical devices when designing systems, configuring, and locating installation of their systems. Moreover, patients and physicians should all be aware of the possibility of adverse effects of implantable neurostimulators from RFID emitters. In the future, our goal is to increase the number and variety of tested implantable neurostimulators and simulate RFID emitters to decrease testing time.

## List of abbreviations

EMC: Electromagnetic Compatibility; RFID: Radio Frequency Identification; FDA: U.S. Food and Drug Administration; ICD: Cardioverter Defibrillator; IPG: Implantable Pulse Generator; DUT: Device Under Test

## Competing interests

The authors declare that they have no competing interests.

## Authors' contributions

OSP, SJS, JWG, DMW have made substantial contributions to the conception and design, acquisition, analysis and interpretation of data. CLS has made substantial contributions in analysis and interpretation of data. All authors were involved in drafting the manuscript. All authors read and approved the final manuscript.

## Disclaimer

The mention of commercial products, their sources, or their use in connection with material reported herein is not to be construed as either an actual or implied endorsement of such products by the Department of Health and Human Services.
